# A role for haemolymph oxygen capacity in heat tolerance of eurythermal crabs

**DOI:** 10.3389/fphys.2013.00110

**Published:** 2013-05-15

**Authors:** Folco Giomi, Hans-Otto Pörtner

**Affiliations:** Section of Integrative Ecophysiology, Alfred Wegener Institute for Polar and Marine ResearchBremerhaven, Germany

**Keywords:** thermal tolerance, eurythermy, oxygen capacity, cardiac performance, aerobic metabolism, Decapoda Brachyura

## Abstract

Heat tolerance in aquatic ectotherms is constrained by a mismatch, occurring at high temperatures, between oxygen delivery and demand which compromises the maintenance of aerobic scope. The present study analyses how the wide thermal tolerance range of an eurythermal model species, the green crab *Carcinus maenas* is supported and limited by its ability to sustain efficient oxygen transport to tissues. Similar to other eurytherms, *C. maenas* sustains naturally occurring acute warming events through the integrated response of circulatory and respiratory systems. The response of *C. maenas* to warming can be characterized by two phases. During initial warming, oxygen consumption and heart rate increase, while stroke volume and haemolymph oxygen partial pressure decrease. During further warming, dissolved oxygen levels in the venous compartment decrease below the threshold of full haemocyanin oxygen saturation. The progressive release of haemocyanin bound oxygen with further warming follows an exponential pattern, thereby saving energy in oxygen transport and causing an associated leveling off of metabolic rate. According to the concept of oxygen and capacity limited thermal tolerance (OCLTT), this indicates that the thermal tolerance window is widened by the increasing contribution of haemocyanin oxygen transport and associated energy savings in cardiocirculation. Haemocyanin bound oxygen sustains cardiac performance to cover the temperature range experienced by *C. maenas* in the field. To our knowledge this is the first study providing evidence of a relationship between thermal tolerance and blood (haemolymph) oxygen transport in a eurythermal invertebrate.

## Introduction

Temperature defines the limits of marine animal distribution and shapes the level of various performances (Huey and Stevenson, 1979; Huey and Kingsolver, 1989; Pörtner, 2001; Hochachka and Somero, 2002; Angilletta, 2009). According to the recent concept of oxygen and capacity limited thermal tolerance (OCLTT) each species displays a range of tolerance, the thermal window, characterized by the optimal temperature and various limiting thresholds, two pejus temperature (Tp) and two critical edges (Pörtner, 2001, 2002, 2010). The capacity of oxygen provision to tissues is seen as the primary factor limiting thermal tolerance, especially in the warmth (Pörtner, 2001, 2002). The breadth of this performance range influences the individual capability to cope with acute changes in environmental temperature. Eurytherms that inhabit environments characterized by large and rapid (daily) temperature fluctuations maintain high functional capacities over a wide thermal range. Moving from the optimal temperature toward the edges of the tolerance window, between the warm and the cold pejus ranges, a series of acute physiological mechanisms supports oxygen supply, matching demand and, thereby, the maintenance of performance. Crossing the specific pejus limits of individuals of a species could lead to ecosystem level consequences due to falling performance and loss in abundance (Pörtner and Knust, 2007). At the extreme end of the tolerance range where performance ceases, critical temperatures determine the failure of such compensatory mechanisms leading to anaerobic metabolism and, ultimately, organism collapse and mortality due to the progressive development of cellular stress and irreversible damage, such as protein denaturation (Pörtner, 2001, 2002, 2010). The thermal envelope including these limiting temperatures may shift due to metabolic acclimatization but such acclimatization capacity may be limited.

Thermal tolerance is realized through modified functions at all levels of biological organization: from whole organism (e.g., adjustment of blood circulation and ventilation rate) to tissues (e.g., plasticity of mitochondrial capacity), and molecules (e.g., expression of chaperones) (Somero et al., 1996; Pörtner, 2001, 2010; Fangue et al., 2009). Beyond the optimal range, warming causes a reduction of oxygen availability to tissues and constrains cellular aerobic metabolism. The augmented demand, mainly in mitochondria, exceeds the rate of oxygen supply, and at the critical temperature (Tc), is increasingly covered by anaerobic metabolism leading to end products such as succinate and L-lactate (Zielinski and Pörtner, 1996; Sommer et al., 1997; Pörtner et al., 1999; van Dijk et al., 1999; Frederich and Pörtner, 2000; Peck et al., 2004; Verberk et al., 2013). Within the thermal range the oxygenation levels of arterial and venous blood are maintained supporting the sustenance of functions. Such levels decrease toward thermal extremes and can therefore be adopted as proxies of aerobic scope. Increased ventilation in vertebrates, like *Gadus morhua* (Sartoris et al., 2003) and *Pachycara brachycephalum* (Mark et al., 2002), and invertebrates, such as *Maja squinado* (Frederich and Pörtner, 2000), *Sepia officinalis* (Melzner et al., 2006a,b) and *Arenicola marina* (Schröer et al., 2009) contributes to sustain aerobic scope. Similarly, increased cardiac frequency in fish (Heath and Hughes, 1973; Mark et al., 2002; Farrell, 2009; Farrell et al., 2009) and, in combination with variations of stroke volume, in crustaceans (Spaargaren, 1974; Frederich and Pörtner, 2000; Walther et al., 2009), maintains oxygen delivery to tissues when demand increases but, in turn also contributes to the demand. It has been documented that in fishes cardio-circulation becomes limiting first (Sartoris et al., 2003), while both circulation and ventilation may become limiting in crustaceans (Frederich and Pörtner, 2000).

The present study investigates the window of thermal tolerance in a highly eurythermal species, the green crab, *Carcinus maenas* (Linnaeus, 1758). It aims to identify the mechanisms setting the dimensions of the thermal window by analyzing the physiological processes involved in supporting aerobic metabolism and scope. *Carcinus maenas* lives in a highly variable marine environment, from shallow waters to the intertidal zone. A number of earlier studies have investigated the responses of this species to several environmental parameters, such as oxygen availability (Taylor and Butler, 1973; Taylor, 1976, 1982; Taylor et al., 1977; Hill et al., 1991); salinity (Taylor et al., 1977; Zanders, 1980; Siebers et al., 1982; Ameyaw-Akumfi and Naylor, 1987; McGaw and Naylor, 1992); air exposure (Perkins, 1967; Crothers, 1968; Newell et al., 1972); temperature (Broekhuysen, 1936; Eriksson et al., 1975; Klein Breteler, 1975; Taylor et al., 1977; Taylor and Wheatly, 1979; Cuculescu et al., 1998; Bartolini et al., 2013) pollutants (Bamber and Depledge, 1997; Hebel et al., 1999; Coelho et al., 2008), and *C. maenas* has been considered a reliable model of ectotherm phenotypic plasticity. Thus, to develop a mechanistic explanation of eurythermy we analyzed the integrated responses of respiratory and circulatory systems of the green crab when exposed to acute warming. Consequently, we estimated the roles of oxygen transport processes including haemolymph oxygen carrying capacity by haemocyanin (i.e., the amount of blood-borne oxygen available to whole organism functioning) in this eurythermal species.

## Materials and methods

### Experimental animals and temperature incubation

*Carcinus maenas*, the intertidal green crab, can experience and tolerate acute thermal events, salinity stress, anoxic episodes and air exposure (Crothers, 1968; Newell et al., 1972; Eriksson et al., 1975; Winkler et al., 1988). *C. maenas* caught in the Wadden Sea were obtained from local fishermen at Carolinensiel, Germany, during autumn 2008 and 2009. Being an invertebrate, no specific permits were required. Locations were not privately owned nor protected and this species is not endangered.

Specimens were collected by trawl net on muddy and sandy bottoms, and only adult males were used. During acclimation periods of 1–3 months, the animals were maintained in a 1 m^3^ tank equipped with a re-circulating filter system (protein skimmers, nitrification filters, UV-disinfection units) at the Alfred Wegener Institute, Bremerhaven, Germany. Animals were fed a diet of mussels and clams under a constant dark:light cycle (12·h:12·h) and constant temperature regime (10 ± 0.1°C). Salinity was maintained between 32 and 35‰, and water pH remained between 8.0 and 8.2.

The experimental protocol was identical between experiments. Animals were starved for 72 h and then transferred to the experimental set-up. Crabs were immobilized on a plastic grid by fixing chelae and walking legs with cable tie. After preparation, all the specimens were acclimatized for 16–24 h before the start of the experiments. This protocol allowed the animals to stabilize in resting conditions which were experimentally verified by checking the stability of the signal before starting the thermal ramp. No animal death or leg detachment was recorded during the preparatory phases or during the experiments. Different groups of crabs were used to assess cardiac activity, respiration and ventilation rates, and the dissolved oxygen patterns.

The experimental set up comprised an insulated bath of continuously aerated seawater (70 × 50 × 40 cm) immersed in a larger bath (90 × 60 × 50 cm) that was connected to a thermostat (Integral T 1200, Lauda, Lauda-Königshofen, Germany). The use of two baths supported a linear warming trend and the maintenance of high seawater quality. Temperature, starting from the control (10°C), was changed in a stepwise procedure at an average rate of 1°C·h^−1^ and was maintained constant during measurements.

### Cardiac activity

**Figure 1:**
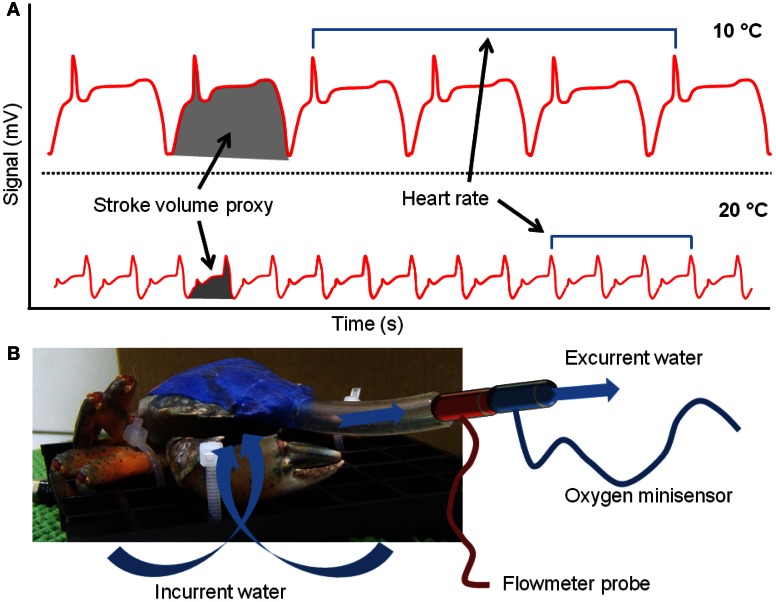
**(A)** Representative traces of the photoplethysmograph signal of cardiac activity obtained from a specimen of *Carcinus maenas* at 10 and 20°C. The integral of the signal was adopted to estimate a proxy for stroke volume (SVP, gray areas) while heart rate was computed from the frequency of the signal (blue bars). **(B)** Schematic of the “rubber mask” device used to simultaneously measure ventilation effort and oxygen consumption rate (shown on a crab temporarily emersed from its water bath).

Heart rate was recorded using the non-invasive photoplethysmograph technique introduced by Depledge (1984). The photosensor (isiTEC, Bremerhaven, Germany) was placed on the carapace of the crabs above the pericardial sinus and firmly fixed using cyanoacrylate glue and dental periphery wax. The photosensor and temperature probe were connected to a computer system for data recording by use of CHART 4 (PowerLab, AD Instruments, Australia). Acute temperature dependent heart rate was calculated as the average over 2 min data recording periods, every 0.2°C. The dimension of the photoplethysmographic signal has a linear relationship with volumetric quantities (Johansson and Öberg, 1999a,b). Accordingly, photoplethysmography is applied to monitor respirometric and cardiac parameters, such as respiration volume, stroke volume, cardiac output, blood pressure, and related pathologies in clinical measurements (for review see Allen, 2007). For measurements performed on human subjects, signal integrals are generally transformed to define quantities through conversion factors. Adopting this rationale, we implemented a procedure to estimate variations in stroke volume and cardiac output for *Carcinus maenas*. Since conversion factors have not been estimated in crabs, we assumed that the integral of the photoplethysmographic signal represents a proxy for stroke volume (SVP, stroke volume proxy). SVP was computed by calculating the average integrals of the signal from 10 strokes, every 0.4°C (Figure 1A), and were expressed in arbitrary units. Temperature was constant during each measurement period. Because of the different thicknesses of the carapace and slightly different locations of the photosensor, data from different individuals are not comparable in absolute values. Thus, all the data points were transformed to percent fractions of the values at the beginning of the experiment (10°C) and thereby represent the relative change of SVP (Figure 1A). This procedure allowed us to quantify the relative change of the signal during the thermal ramp. A proxy for cardiac output was obtained by multiplying heart rate and SVP, and was expressed in arbitrary units.

### Respiration and ventilation rate

To simultaneously measure ventilation effort and oxygen consumption, a “rubber mask” was created and modified from Johansen et al. (1970). In detail, a piece of latex dam was glued on the anterior ventral part of the carapace, including the maxillipedes, but not covering the lower opening of the branchial chamber above the chelae. This assured unrestrained water flow into the gill chamber. A small hole was made in the dam at the level of the higher opening of the chamber, just below the eyes. A 5 cm length (inner diameter 5 mm) Tygon tube (Saint-Gobain Performance Plastics) was inserted within this hole and glued with cyanoacrylate adhesive. Finally, the dam was glued onto the upper part of the carapace and this sealed the “respiration chamber” (Figure 1B). Oxygen partial pressures of excurrent water were monitored continuously with a Fibox-2 oxygen meter (Presens, Regensberg, Germany) connected to the tube. Oxygen sensitive probes (Minisensor, Presens, Regensberg, Germany) were calibrated prior to each experiment in air-saturated (100%) and in oxygen-free seawater, using 5% w/w sodium diothionite (0%). Water oxygen concentrations were calculated from values of oxygen partial pressure using temperature-dependent solubility coefficients for oxygen (αO_2_, μmol × l^−1^ × Torr^−1^). The volume flow of the excurrent water produced by the ventilation of the branchial chamber was measured using an electromagnetic flow-meter (RT-500, Hugo Sachs Elektronik, March-Hugstetten, Germany; probe head: inner diameter 3 mm, length 10 mm) connected to the tube, next to the oxygen optodes. The flowmeter was calibrated at the experimental temperature by means of a peristaltic pump. Preliminary tests showed that the ventilation volume generated by the crabs ranges between 2 and 50 ml × min^−1^, thus these extreme values were adopted for each calibration. The optode-flowmeter setup allowed for the concomitant measurement of oxygen uptake, calculated by multiplying the difference between the oxygen concentration in the water bath and excurrent flow by ventilation volume. The combination of these data also enabled the calculation of oxygen extraction efficiency (OEE, expressed in volume of consumed oxygen per minute divided by the volume of oxygen crossing the respiratory epithelium per minute), which represents the effective oxygen extraction from a certain volume of water and indicates the capacity to maximize oxygen uptake.

To ensure that measurements collected with the “masking” procedure reflect the natural values of unrestrained resting animals, ventilation volumes were checked at the beginning of each experiment. It is worth mentioning that the ventilation activity was a very sensitive parameter in decapod crustaceans, and precisely reflects their metabolic status and their physiological conditions (Jouve-Duhamel and Truchot, 1985; Wilkens et al., 1985). With the use of long term laboratory acclimated crabs (more than 1 month) fully recovered from the handling stress (more than 5 h), ventilation flow volumes in *C. maenas* naturally decreased to an undisturbed resting steady-state of 7–10 ml × min^−1^ (Arudpragasam and Naylor, 1964). Our maximum care to work with fully acclimated and unstressed animals in fact reproduced these low ventilation flows of 7–10 ml × min^−1^. In contrast, excited animals acclimatized to experimental conditions for only 30 min are characterized by high ventilation flows between 30 and 70 ml × min^−1^ (Arudpragasam and Naylor, 1964).

### Dissolved oxygen measurements

Measurements of arterial and venous *P*O_2_ were carried out with microoptodes (NTH-PSt1-L5lTF-PC3,1-NS 35x1,20-YOP, PreSens GmbH, 93053 Regensburg, Germany). Data were recorded on-line with TX2-A oxygen meters and software (Oxy View TX2 C 4.02) (PreSens Regensburg, Germany), which include internal algorithms to compensate for temperature changes during the reading. Prior to each experiment, optodes were calibrated in air-saturated seawater (100%) and in oxygen-free seawater, using sodium dithionite (0%). Prior to surgery, the needle was rinsed with a heparin solution (5000 U/ml) to prevent hemolymph clotting around the oxygen probes. To measure the oxygen saturation of post-branchial blood, the tip of the needle was inserted through a hole (maximum width 0.2 mm) drilled into the carapace above the pericardial sinus, avoiding injury to the hypodermis. Pre-branchial blood was probed by inserting the needle into the arthrodial membrane at the base of the fourth or fifth pereiopod in order to reach the venous sinus. In both cases, cyanoacrylate adhesive was used to seal the hole, to prevent hemolymph loss and maintain the probe firmly fixed during the measurements. Oxygen values were recorded as % air saturation and converted to *P*O_2_ and [O_2_].

### Assessment of haemolymph oxygen transport

To evaluate the relative contribution of dissolved and pigment bound oxygen to oxygen transport within the experimental temperature range, data from the present study were integrated with results on oxygen binding to *Carcinus maenas* haemocyanin reported by Weber et al. (2008). The use of literature data on haemocyanin functioning instead of direct measurements was justified because the variations in haemolymph *P*O_2_ (reflecting the fraction of physically dissolved blood oxygen content) cover a much greater range than the *P*O_2_ range of haemocyanin functioning (fraction of blood oxygen content bound to haemocyanin). Thus, we computed the contribution of haemocyanin to oxygen delivery through the implementation of data from Weber et al. (2008) using the oxygen partial pressure data we produced. This implies a simple calculation of the amount of oxygen released with changing *P*O_2_ from a certain concentration of haemocyanin. To determine the change in percent oxygen saturation the calculation was performed by interpolating the venous partial pressures measured *in vivo* on the oxygen binding curves. In detail, P_50_ values under different experimental conditions and oxygen saturation sigmoidal curves at 10 and 20°C were acquired from Figures 3, 4, respectively, of Weber et al. (2008). In *C. maenas*, the pH of prebranchial haemolymph in animals acclimated at 10°C was pH 7.8 and varies with temperature at a rate of −0.016 pH unit × °C^−1^ (Truchot, 1973). Thus, during the acute temperature ramp between 10 and 25°C we estimated a decrease of haemolymph pH to 7.56. At this pH, temperature reduces haemocyanin oxygen affinity at a rate of 0.26 mmHg × °C^−1^, increasing P_50_ to up to 18 mmHg at 25°C. Any interfering effect of lactate, which increases haemocyanin affinity at 10°C, becomes negligible at 20°C (Weber et al., 2008). At extreme temperatures (not covered by Weber et al.'s data), changes in haemocyanin oxygen affinity by allosteric modulation and Bohr shift may maximize capillary oxygen release, but the bulk of oxygen has been released at lower temperatures such that the picture of how the release of oxygen supports heat tolerance in *C. maenas* is not modified. Thus, the percentage of haemocyanin oxygen saturation, and consequently, the amount of oxygen released, was calculated for each 0.5°C warming, using the oxygen partial pressure measured *in vivo*, and a combination of haemocyanin oxygen saturation curves matching the experimental conditions applied in the present study. Results are reported for three hemocyanin concentrations, in order to consider its natural variation (Truchot, 1978; Boone and Schoffeniels, 1979).

### Data analyses and statistics

Discontinuities in the slopes of heart rate and SVP changes with temperature were calculated from intersections of fitted two-phase regressions according to the minimum sum of squares. Simple linear and exponential regression analyses were performed using SigmaPlot 11.0 (SSPS Inc., Point Richmond, CA, USA). Non-linear regression curves were fitted using exponential growth (*y* = *y*_0_ + *ae*^*bx*^) or exponential rise to maximum [*y* = *y*_0_ + *a*(1 − *e*^−*bx*^)] equations. Since data are dependent (measures were performed on the same individuals across the temperature ramp) ventilation volume, oxygen consumption and OEE at different temperatures were compared using analysis of variance for repeated measures. If the overall tests were significant, pairwise comparisons were performed using the *t*-test for dependent data and applying the Bonferroni correction for multiple comparisons (significance level: 0.05/15 = 0.0033). For analyses of dissolved oxygen content, means were compared using ANOVA and Dunnet's *post-hoc* test was applied to identify differences from the acclimation temperature (control, 10°C). All data are presented as means ± s.e.m.

## Results

### Cardiac activity

**Figure 2:**
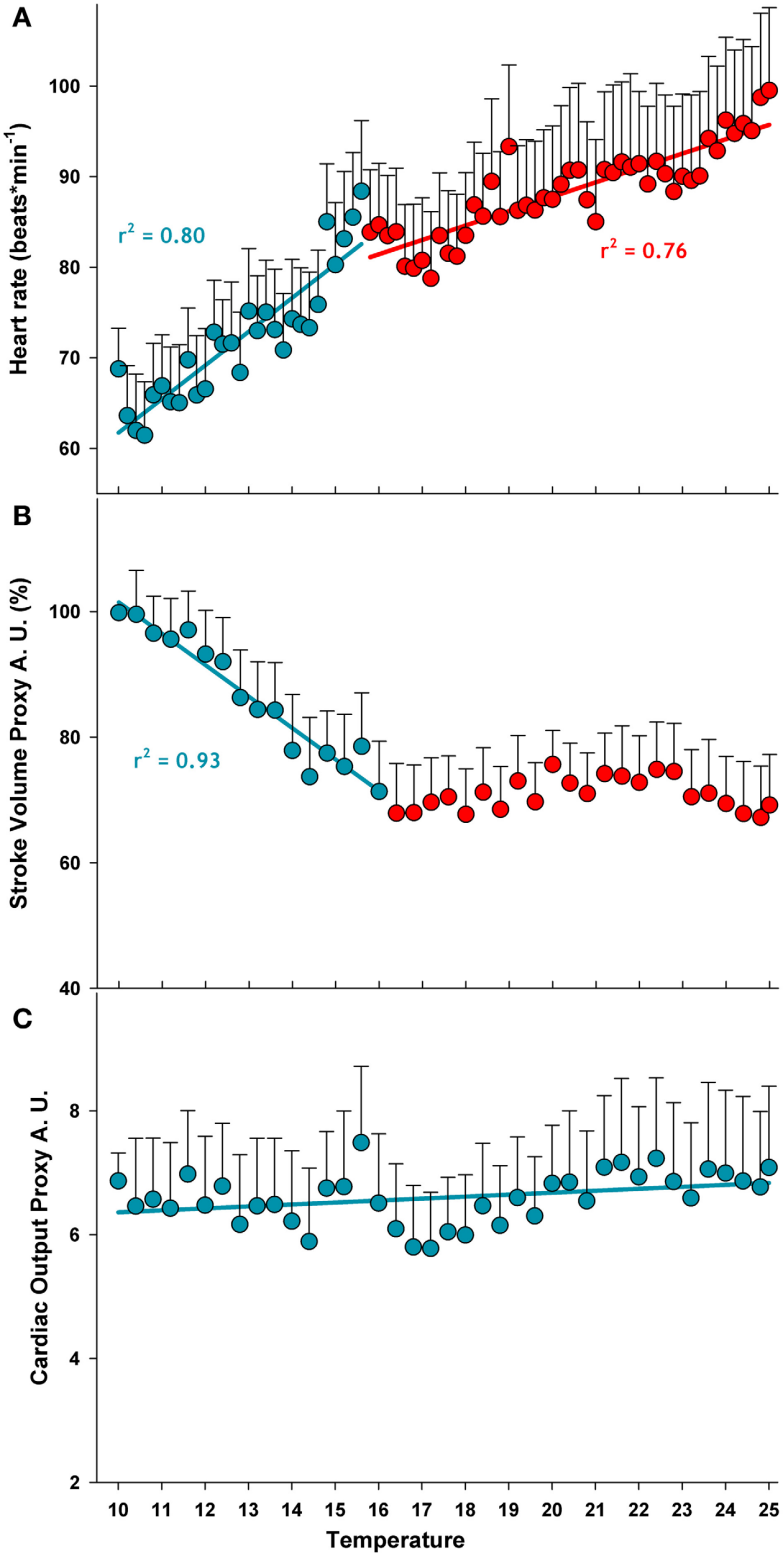
**Temperature dependent pattern of cardiac activity during acute warming from 10 to 25°C.** The two colors represent the discontinuities in the temperature dependence of heart rate and SVP data (means) analyzed from linear regressions, intersecting at the respective breakpoints. Regressions for heart rate data (panel **A**) are: *y*_(10-15.6°C_) = (24.6 ± 4.6) + (3.717 ± 0.345)*x*, *p* < 0.0001; *y*_(15.8-25°C_) = (56.0 ± 2.7) + (1.590 ± 0.131)*x*, *p* < 0.0001. Regressions for SVP (panel **B**) are *y*_(10-15.6°C_) = (151.6 ± 5.4) + (−5.011 ± 0.417)*x*, *p* < 0.0001; *y*_(16-25°C_) = n.s. The discontinuities in the slopes are at 15.6°C, for both parameters. The regression for the cardiac output proxy (heart rate x SVP) (panel **C**) was not significant, indicating that cardiac output was constant over the whole temperature range. Data are means ± s.e.m., *n* = 11.

Heart rate increased and SVP fell during acute warming. Mean heart rate ranged from 62 to 100 beats × min^−1^ between 10.2 and 25°C (Figure 2A). Heart rate increased more steeply between 10 and 16°C, as revealed by the two-phase regression, while SVP fell. During further warming, heart rate increased less rapidly and the volume generated per heart contraction remained unchanged (Figure 2B). From these results a common response of heart activity to warming became evident: in animals acclimated to 10°C both parameters were strictly correlated and share a common breakpoint at 15.6°C beyond which heart rate and SVP level off. However, to better describe temperature dependent heart activity, cardiac output was estimated as the volume pumped by each contraction multiplied by heart rate (Figure 2C). There was no significant change in cardiac output between 10 and 25°C, indicating that the stroke volume decreased with rising frequency. Thus, even if pronounced changes occurred in the pattern of cardiac performance, the total effort of cardiac activity remained stable.

From a methodological perspective, the implementation of the analytical procedure developed in clinical physiology has provided promising results on cardio-circulatory data for crabs and their relative changes with temperature. For further applications of this analysis, the estimation of stroke volume from photoplethysmograph signal could be cross validated with complementary tools like the pulsed-Doppler flow-meter.

### Aerobic metabolism

**Figure 3:**
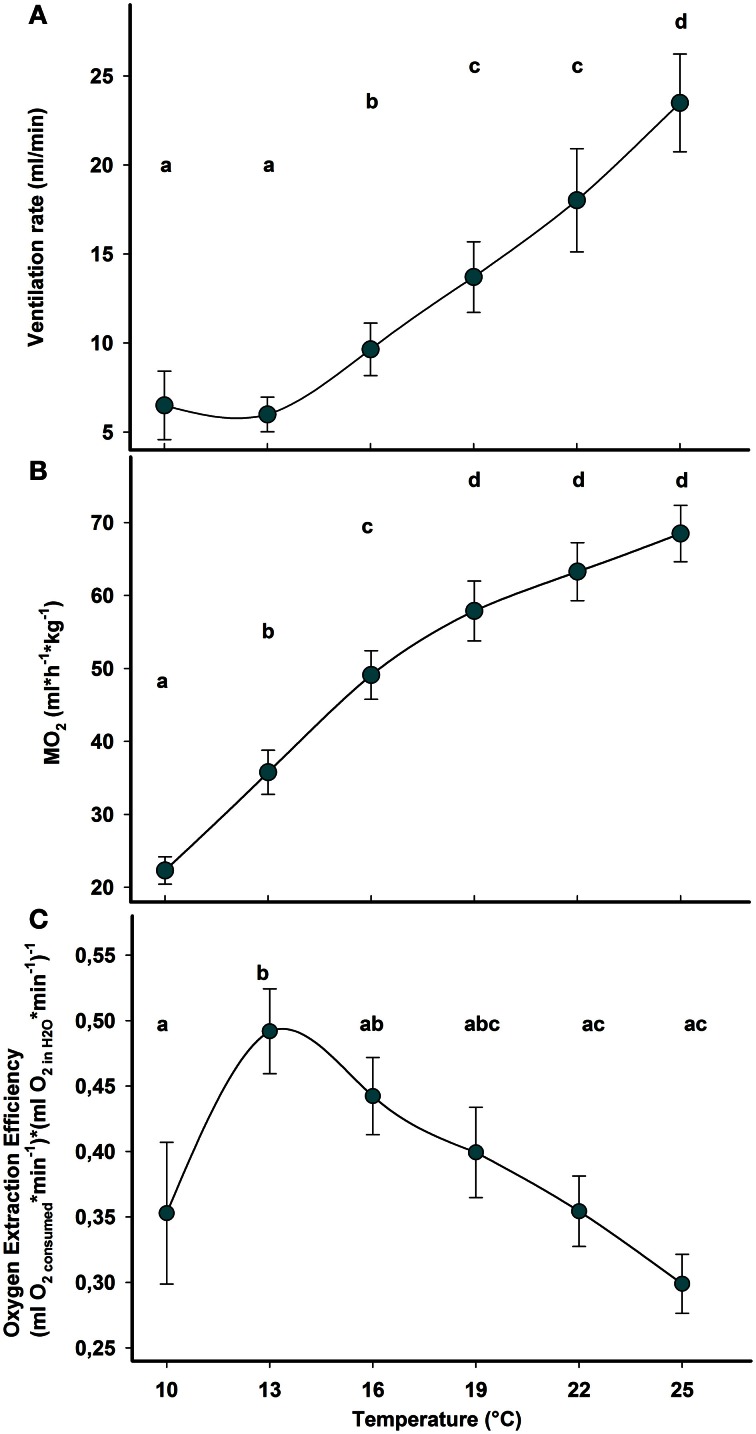
**Temperature dependent pattern of aerobic metabolism and ventilation during acute warming from 10 to 25°C. (A)** Water flow expressed in volume of excurrent flow × minute^−1^. **(B)** Oxygen consumption rate (MO_2_). **(C)** Oxygen extraction efficiency (OEE) from ventilation current. Within each panel, different letters indicate statistically significant differences between treatments (Student–Newman–Keuls test; *p* < 0.05). Data are means ± s.e.m., *n* = 10.

**Figure 4:**
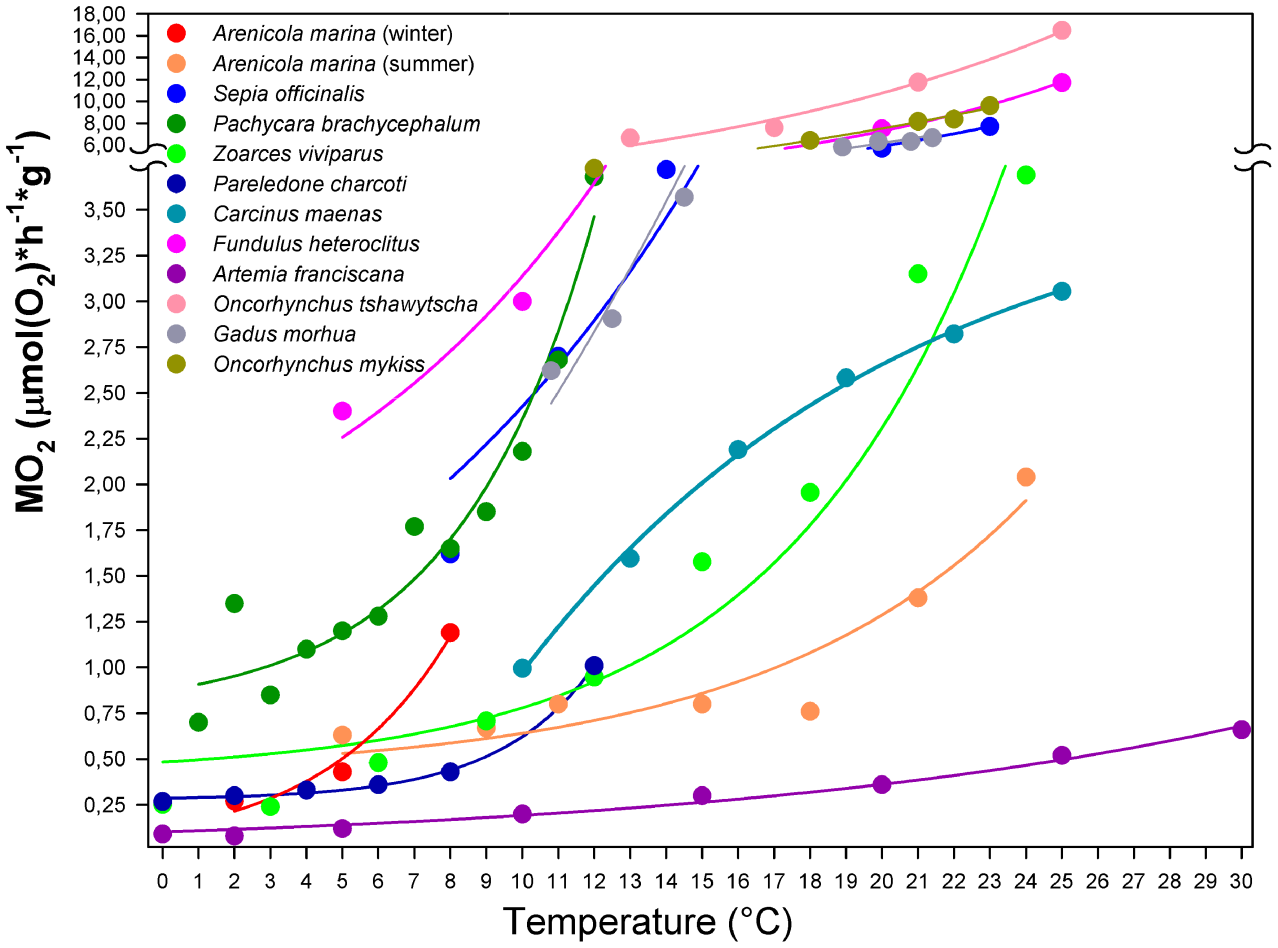
**Temperature-dependent rate of resting oxygen consumption for marine ectotherms.** Typical metabolic trends reported for marine ectotherms match exponential curves turning into an asymptote, while approaching a maximum rate at a temperature, which likely corresponds to the species-specific critical temperature (Tc, as defined by the OCLTT concept). Upon further warming, metabolism decreases rapidly reflecting a failure in sustaining metabolic oxygen demand (data not shown). The temperature dependent oxygen consumption rate of *Carcinus maenas* (and possibly, other eurytherms) deviates from this general model. Non-linear regression fits the data better with an exponential rise to a maximum [equation: *y* = −3.315 + 7.052(1 − *e*^−0.094*x*^), *r*^2^ = 0.99, *p* < 0.001]. This curve approaches the asymptote of 3.74 [μmol(O_2_) × h^−1^ × g^−1^], suggesting enhanced efficiency in metabolic maintenance over a wide temperature range and revealing no evidence of critical temperature at the level of oxygen demand. Data from: Wittmann et al., 2008, *Arenicola marina*; Melzner et al., 2006b, *Sepia officinalis*; Mark et al., 2002, *Pachycara brachycephalum*; van Dijk et al., 1999, *Zoarces viviparus*; Pörtner, 2001, *Pareledone charcoti*; Fangue et al., 2009, *Fundulus heteroclitus*; Irwin et al., 2007, *Artemia franciscana*; Clark et al., 2008, *Oncorhynchus tshawytscha*; Keen and Kurt Gamperl, 2012, *Oncorhynchus mykiss*; Gollock et al., 2006, *Gadus morhua*.

Ventilation and respiration rates increased during acute warming in *Carcinus maenas*. Ventilation rate showed significant differences between temperatures (*F*_(6, 4)_= 43.67; *P* = 0.001), with rates significantly higher than the control above 16°C (Figure 3A). Oxygen consumption rate increased significantly during warming (*F*_(6, 4)_= 175.06; *P* < 0.0001) but the increment was larger between 10 and 16°C. At 19°C and above, the test did not reveal significant changes suggesting a biphasic response of aerobic metabolism: an initially strong increase of respiration rate, which tended to stabilize during further warming (Figure 3B). This finding was further supported by the regression of oxygen demand changing with temperature. Non-linear regression with an exponential rise to a maximum explains the data, over the whole range, better than an exponential growth curve: *y* = −3.315 + 7.052(1 − *e*^−0.094*x*^), *r*^2^= 0.99, *p* < 0.001, (for further details and explanation see Figure 4). Oxygen consumption and water flow through the gill chamber yields OEE and its temperature dependence (Figure 3C). The overall results indicate that temperature has a significant effect on the OEE [*F*_(6, 4)_= 22.49; *P* = 0.005]. Interestingly, OEE significantly increased at the first step of warming (13°C), while no further differences between values along the thermal ramp and the control (10°C) were detected (Figure 3C).

### Oxygen transport in haemolymph

**Figure 5:**
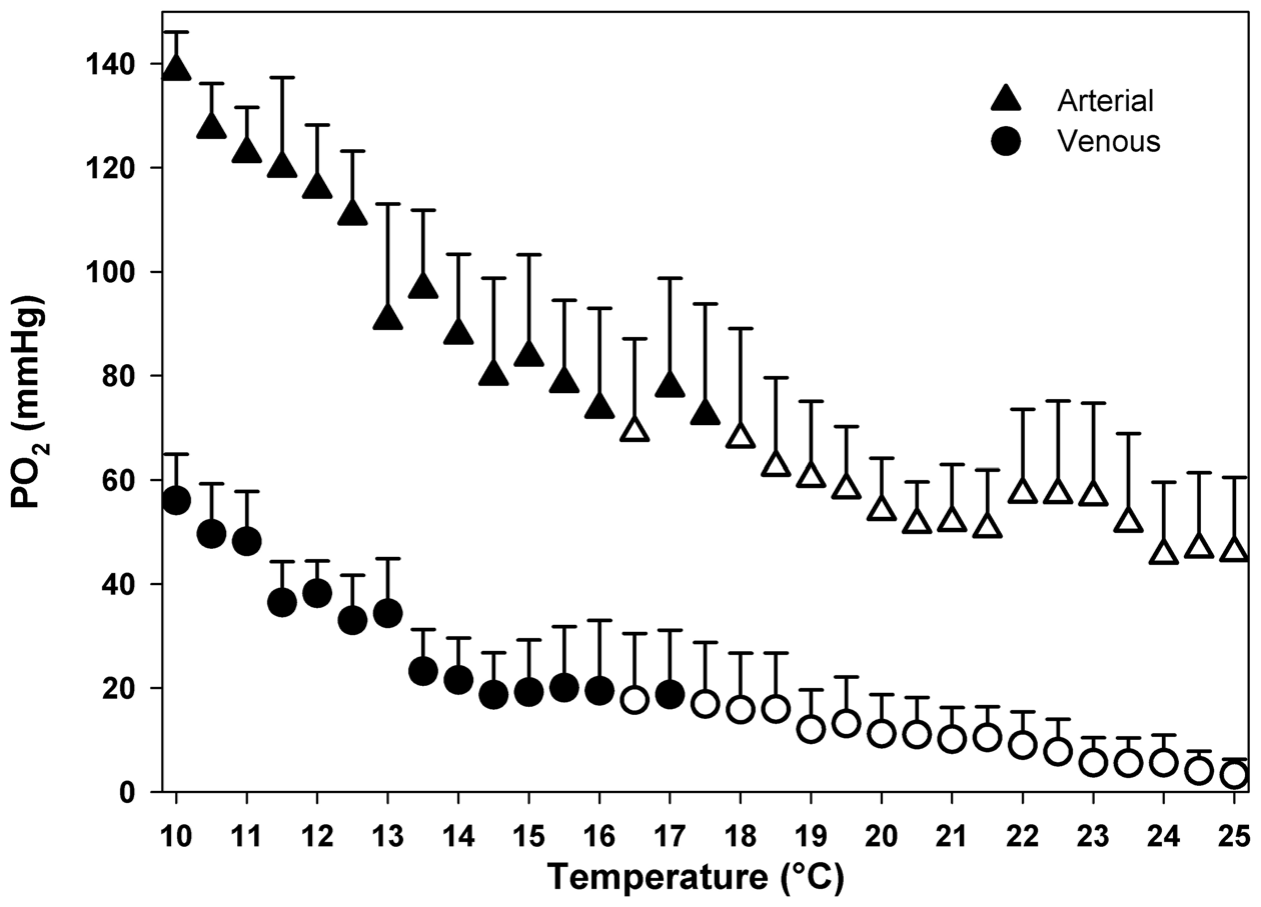
**Temperature dependent patterns of arterial and venous oxygen partial pressure, during acute warming from 10 to 25°C.** Open symbols denote values that are significantly different from the respective controls, Dunnett's test (*P* < 0.05). Data are means ± s.e.m., *n* = 4.

Temperature dependent changes in the partial pressure of oxygen are shown in Figure 5, reflecting the changes in physically dissolved oxygen levels. A marked reduction in oxygen partial pressures occurred in arterial and, consequently in venous blood at higher temperatures. Arterial oxygen tensions decreased from 138 to 46 mm Hg, while venous values ranged from 56 to 3 mm Hg. Compared to the control, significant changes in oxygen partial pressure occurred at 16.5°C in both arterial and venous compartments [Dunnett's test: arterial: *F*_(30, 93)_= 2.91; *P* < 0.0001; venous: *F*_(30, 93)_= 2.58; *P* < 0.001]. The major change in both arterial and venous oxygen partial pressures occurs during the initial warming phase with a rapid fall of about 40 mm Hg between 10 and 14°C (Figure 5).

**Figure 6:**
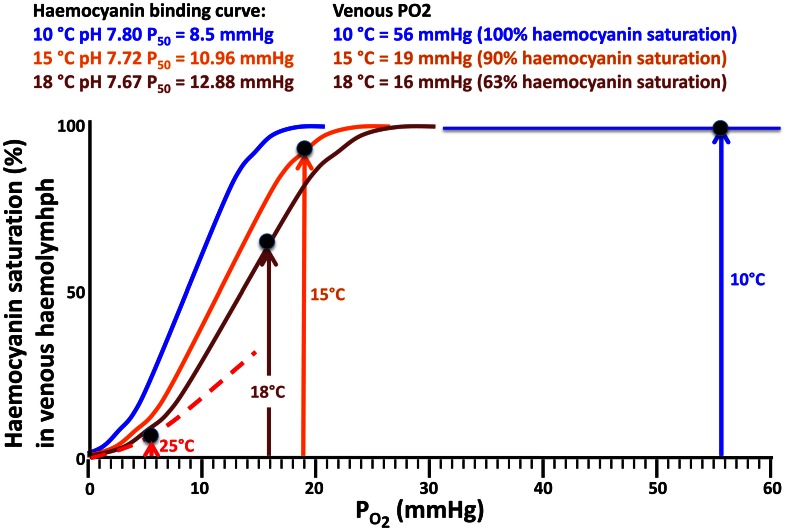
**Temperature dependent oxygen binding curves and haemocyanin oxygen saturation levels (data from Weber et al., 2008) resulting from venous *P*O_2_ data (Figure 5), thereby illustrating how venous oxygen saturation develops with warming.** For further explanations see Materials and Methods.

**Figure 7:**
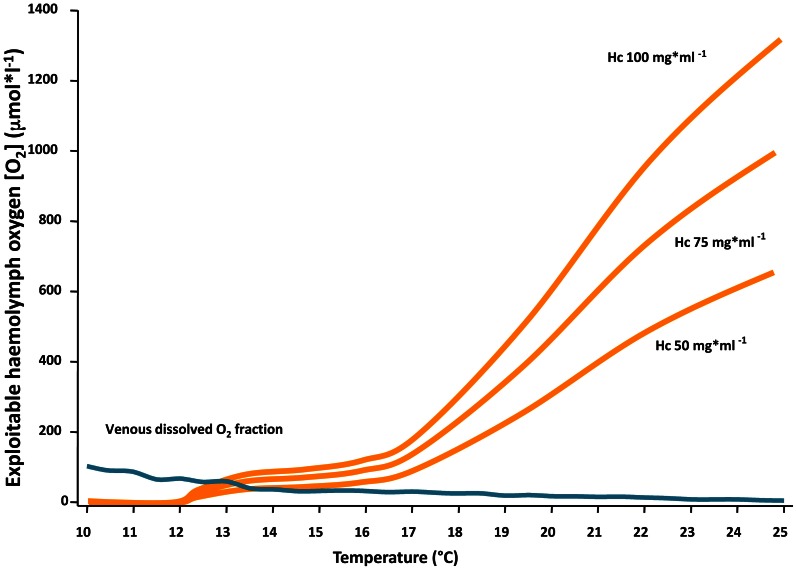
**Contribution of the physically dissolved fraction of oxygen (blue line) and the amount released by haemocyanin (Hc) in the venous blood (orange lines) to cover whole animal oxygen demand at various temperatures.** Note that under control conditions and moderate warming (10–13°C) haemocyanin remained fully saturated and did not contribute to oxygen delivery. Between 13 and 18°C, when venous *P*O_2_ decreased significantly, haemocyanin started to release oxygen and compensated for the reduction of dissolved oxygen levels. At the highest temperature (18–25°C), when the venous dissolved O_2_ fraction approached zero, haemocyanin continued to unload oxygen, resulting in a maximized contribution to oxygen transport and aerobic metabolism. The three curves track the natural and thus optional variation in haemocyanin concentration. This mechanism was also effective at high temperature because haemocyanin oxygen saturation at the gills was efficiently sustained over the entire ramp as indicated by the maintenance of arterial *P*O_2_ values at saturating levels (for oxygen concentration in arterial blood cf. Figure 5). For further explanations see Materials and Methods.

The evaluation of haemocyanin oxygen transport during acute warming indicates an increasing contribution of bound oxygen to cover the rising oxygen demand and extend the heat tolerance range. The arterial *P*O_2_ remains high enough to guarantee the complete saturation of haemocyanin across the entire temperature range indicating complete arterial oxygenation of the pigment at the gills (Figure 5). In contrast, during acute warming, the circulating fraction of haemocyanin bound oxygen decreased at the tissue level (driven by the falling venous *P*O_2_) indicating progressive oxygen unloading from the haemocyanin (Figure 6). Specifically, when crabs were at acclimatization temperature and slightly beyond (10–13°C), venous haemocyanin remained fully saturated and did not contribute to oxygen delivery. Upon further warming, haemocyanin started to release oxygen and thereby buffered venous *P*O_2_ and slowed the development of hypoxemia. This release increased exponentially in the highest temperature range (17–22°C) and began to stabilize beyond this temperature, when venous oxygen partial pressure continued to fall at a faster rate (Figure 7).

## Discussion

The OCLTT concept defines the temperature window of a species as a function of the extent and maintenance of its aerobic scope as required for energy allocation to growth, behaviors and immune functions. Especially in the warmth, the deviation from optimum performance and the associated decline of animal fitness beyond pejus limits was directly related to a mismatch in oxygen uptake and demand and progressive hypoxemia, followed by a transition to anaerobiosis at critical limits, involving lactate and succinate accumulation (for review see Pörtner, 2001, 2010). During progressive warming, aquatic ectotherms experience an increasing shortage of oxygen supply to tissues, even at rest (Sommer et al., 1997; Pörtner et al., 1999; van Dijk et al., 1999; Mark et al., 2002; Sartoris et al., 2003; Peck et al., 2004; Melzner et al., 2006a,b; Schröer et al., 2009). In crustaceans, at temperate latitudes, warm induced hypoxemia has been demonstrated in *Maja squinado*, *Cancer pagurus* and *Hyas araneus* (Frederich and Pörtner, 2000; Metzger et al., 2007; Walther et al., 2009). In all cases, a decrease of arterial *P*O_2_ occurred when animals were exposed to warm temperatures beyond the optimal range, likely due to the fact that ATP turnover and associated oxygen demand rose critically upon warming (Pörtner, 2001) leading to a mismatch in oxygen supply and demand. Similarly, our data reveal a clear negative relationship between blood oxygenation and elevated temperature in *Carcinus maenas*. From the present study (Figure 5) venous *P*O_2_ appears as a more adequate indicator of the progressive limitation in oxygen availability to tissues than arterial *P*O_2_. Venous *P*O_2_ directly reflects the amount of oxygen available at tissue level and consequently constitutes the most accurate indicator of an emerging mismatch between oxygen supply and demand. In contrast, arterial *P*O_2_ is defined by the efficiency of arterial oxygen uploading at gas exchange structures.

An increase in metabolic demand during acute warming has been a general phenomenon common to most aquatic ectotherms studied thus far. The oxygen consumption rate rises exponentially along the temperature ramp reaching a critical point beyond which the capacity limit of oxygen supply is reached and the exponential phase ends (Figure 4). In the cephalopod *Sepia officinalis* this point was reached at the Tc when anaerobic metabolism set in (e.g., Melzner et al., 2006a). In fish, the leveling off of the exponential rise in oxygen consumption follows the progressive reduction of physiological capacity (Gollock et al., 2006; Keen and Kurt Gamperl, 2012). Such temperature dependent exponential rise of aerobic metabolic rate was found in animals with extremely contrasting bauplans and lifestyles, e.g., in highly active vertebrates such as salmonids and cod (Gollock et al., 2006; Clark et al., 2008; Keen and Kurt Gamperl, 2012), and in burrowing annelid invertebrates such as *Arenicola marina* (Wittmann et al., 2008). This exponential range can shift as a result of thermal acclimation.

*C. maenas* may deviate from this response model. The initial increase in oxygen consumption stabilized after the first warming steps (10–16°C) and decreased to rates below those expected from a continued exponential pattern. This indicates a reduction of metabolic costs of oxygen supply which allows *C. maenas* to maintain aerobic performance up to higher temperatures. Upon further warming oxygen consumption tended to increase less steeply and in parallel, venous *P*O_2_ showed a shallower decline beyond the 15–16°C threshold seen in animals acclimated to 10°C. The present haemolymph oxygen data indicate a pattern of progressive alleviation of the hypoxemia until the Tc is reached. Thus, aerobic metabolism may have been supported by compensatory mechanisms suitable to delay hypoxemia leading to an extension of the aerobic range and consequently, a wider thermal tolerance window.

To identify the mechanism that contributes to setting the dimensions of the thermal window in this eurytherm, we considered the different elements supporting whole animal oxygen demand. Specifically, we focused on those involved in oxygen transport, in line with the predictions of the OCLTT concept. The maintenance of oxygen supply during acute warming was generally accomplished by an increase of ventilation and cardiac performance. The present data also indicate that oxygen transport under warm conditions increasingly involved the amount of oxygen bounded to haemocyanin. *C. maenas* haemocyanin displays a high oxygen affinity reflected in a low P_50_ of 7.7–7.8 mmHg (1.026–1.039 kPa) at pH 7.83 and 10°C (Weber et al., 2008). Under normoxia and at acclimation temperature the venous *P*O_2_ was around 57 mmHg (Figures 5, 6). Under these conditions the oxygen demand of the animal was covered entirely by the dissolved oxygen fraction as haemocyanin was unable to unload oxygen at this high *P*O_2_, and thus remained permanently oxygenated. Thus, haemocyanin acts as an oxygen store and does not contribute to covering oxygen demand of the resting animal within the optimum temperature range. However, acute warming augmented oxygen demand and decreased venous *P*O_2_ low enough to support oxygen unloading of the haemocyanin (Figures 6, 7). An abrupt fall of oxygen partial pressure occurred between 10 and 14°C while it decreased more slowly thereafter (Figure 5). Thus, in the range 13–18°C, haemocyanin released between 10 and 30% of its bound oxygen in venous blood on each cycle, more than fully compensating for the warming-induced reduction in physically dissolved quantities. Upon continued warming up to 25°C, further oxygen was released following the exponential phase of the binding curve, thereby reducing the circulatory work needed to cover the enhanced oxygen supply. Such was indicated by the decrease in slope of heart rate with increasing temperature beyond 15°C. In turn, the associated energy savings caused by the onset of oxygen release from the haemocyanin minimized the warming induced increase in metabolic rate.

It is interesting to note that OEE, apart from a marked rise during the initial step of warming, remained constant over the entire temperature range. The stabilization of heart rate and SVP following the initial variation indicates a relaxation of the cardiac workload when oxygen demand was progressively covered by haemocyanin oxygen transport. An almost similar biphasic variation of stroke volume during temperature increase has been reported in less eurythermal crabs such as *Cancer magister*. Results showed that a partial stabilization of stroke volume occurred after the initial rapid decline during warming but this adjustment was not sufficient to counterbalance the increasing heart rate (De Wachter and McMahon, 1996). As a result, the cardiac output of C. *magister* generally increased during progressive warming (De Wachter and McMahon, 1996; De Wachter and Wilkens, 1996). In contrast, the proxy for cardiac output remained stable over the entire temperature ramp in *C. maenas*, supporting the efficiency of compensatory oxygen transport and demonstrating that cardiac performance remained unaffected by acute heat stress.

It should be noted that decapod crustaceans adjust heart activity and capacity through nervous and hormonal controls (Wilkens, 1999; McMahon, 2001). In case of *C. maenas* circulatory adjustments during acute warming arguably contribute to minimize the energy expenditure of the heart while maintaining effective performance and a wider thermal window through enhanced involvement of blood oxygen transport. As ventilation rate does not benefit from the involvement of haemocyanin, it continued to increase during the thermal ramp, as indicated by an increased water flux beyond 16°C, while no variation was visible at the beginning of the warming procedure. Thus, animals enhanced water flux through the branchial chamber to facilitate oxygen supply, while the haemocyanin-stored amount begins to be exploited, balancing the workload of the heart.

**Figure 8:**
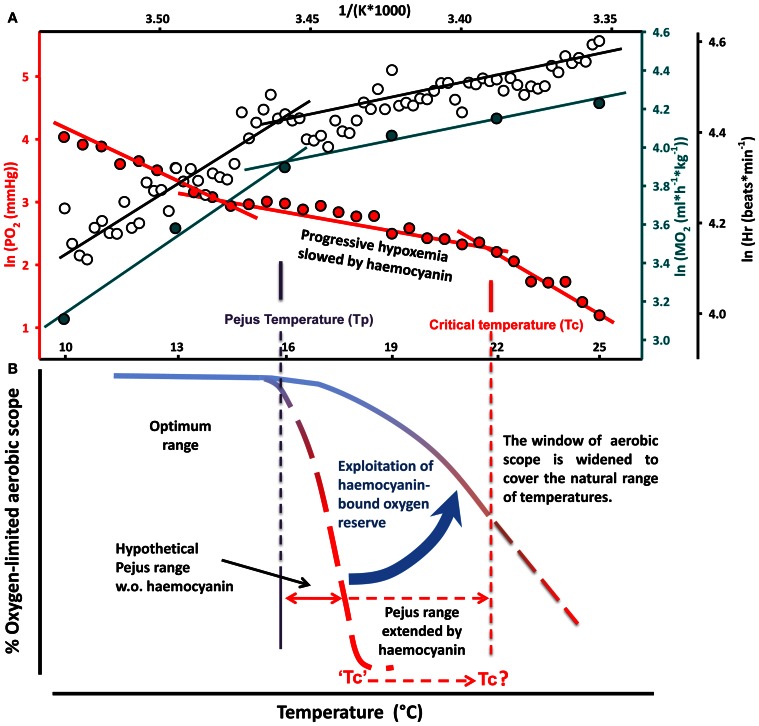
**Conceptual model of the role of haemolymph oxygen capacity in the thermal tolerance of a eurythermal crab. (A)** Arrhenius plot of the temperature dependent changes in oxygen consumption, heart rate and venous *P*O_2_ reveal breakpoints identified as pejus temperature (Tp, left) and critical temperature (Tc, right). **(B)** In aquatic ectotherms acute warming beyond the optimal range causes a progressive mismatch between oxygen supply and demand, thereby limiting aerobic scope and associated functional capacity and performance (Pörtner, 2002, 2010). Widened windows of tolerance in the eurythermal crab are sustained through the exploitation of the haemocyanin-bound oxygen reserve. Compared to a hypothetical animal that lacks haemocyanin, transition to passive tolerance can be delayed and the Tc shifts to its present value. The release of transported oxygen expands the temperature range where aerobic scope is availably due to energy savings in oxygen supply systems and the associated reduction in oxygen demand at high temperatures.

Compared to a hypothetical animal that lacks haemocyanin, these energy savings are expected to shift the upper Tc to higher values (Figure 8B). It is worth mentioning that warming combined with the Bohr effect decreased oxygen affinity (increased P_50_) up to 18 mmHg, thereby facilitating oxygen release during warming. The positive allosteric regulation of haemocyanin by L-lactate which would counteract and reduce oxygen unloading, does not occur at 20°C in the *in vivo* pH range of 7.4–7.8 (Weber et al., 2008).

In conclusion, these results demonstrate for the first time that the ability to progressively enhance the exploitation of haemocyanin oxygen transport sustained aerobic metabolism, saved energy and thereby widened the range of thermal tolerance. The sigmoidal shape of the hemocyanin oxygen binding curve, reflecting the cooperative binding of oxygen, may in fact explain the temperature dependent transition phases in the oxygen consumption curve and in venous *P*O_2_. This is best illustrated in an Arrhenius-plot (Figure 8A), where heart rate and *M*O_2_ increased linearly and steeply during the initial phase of warming when haemocyanin contribution to oxygen supply was minimal. During further warming the release of haemocyanin bound oxygen rose exponentially improving the oxygen transport efficiency of circulation, which in turn reduces the demand to increase circulation effort as is reflected in a lower increment in heart rate and MO_2_. In line with the OCLTT concept, the discontinuities in metabolic rate, heart rate and venous *P*O_2_ (left) characterize the Tp. Any difference between the breakpoints in oxygen consumption, heart rate and venous *P*O_2_ might be explained by the fact that venous *P*O_2_ is shaped by the oxygen demand covered locally via the peripheral vascular system, whereas whole animal oxygen consumption and heart rate reflect the oxygen requirements of the whole organism. The temperature range of reduced slopes in metabolic rate, heart rate and venous *P*O_2_ represents the pejus range where oxygen availability to the organism and thus the aerobic scope for increasing performance became progressively reduced (Figure 8A). The falling partial pressures of oxygen in the haemolymph as well as the decreasing reserve of venous haemocyanin bound oxygen indicate in fact that aerobic scope continued to decline during warming but this decline was minimized when haemocyanin oxygen transport became involved. We postulate that haemolymph *P*O_2_ decreased more slowly in the presence than in the absence of an oxygen binding pigment. The second, right-hand Arrhenius breakpoint (Tc) in the temperature-dependent decline of venous *P*O_2_ would indicate where increasing metabolic demands caused *P*O_2_ to fall below the minimum pressure head of oxygen diffusion to mitochondria although oxygen supply from haemocyanin still remained effective (Figure 8B). The pejus range would thus be extended and the transition to passive tolerance delayed by the involvement of haemocyanin oxygen transport until the Tc was reached.

The present findings cast new light on the role of haemocyanin in the thermal tolerance of *C. maenas*. Haemocyanin bound oxygen becomes more and more involved in covering the oxygen demand of *C. maenas* at the border of its natural temperature range, thereby reducing energy expenditure and extending the thermal tolerance range. Our data provide first evidence for a crucial role of blood oxygen transport in setting thermal windows wide as in eurytherms. These findings emphasize that further investigations of thermal tolerance are needed at all levels of the oxygen transport system. It is reasonable to expect that aquatic, and perhaps terrestrial, ectotherms inhabiting thermally unstable environments have evolved similarly integrated mechanisms including blood oxygen transport to cope with the oxygen and capacity limitation of thermal tolerance.

### Conflict of interest statement

The authors declare that the research was conducted in the absence of any commercial or financial relationships that could be construed as a potential conflict of interest.
